# Application and effects of fever screening system in the prevention of nosocomial infection in the only designated hospital of coronavirus disease 2019 (COVID-19) in Shenzhen, China

**DOI:** 10.1017/ice.2020.119

**Published:** 2020-04-13

**Authors:** Ting Huang, Yinsheng Guo, Shaxi Li, Yanqun Zheng, Lin Lei, Xianhu Zeng, Qiao Zhong, Yingxia Liu, Lei Liu

**Affiliations:** 1 Department of Healthcare-Associated Infection Management, National Clinical Research Center for Infectious Diseases, Third People’s Hospital of Shenzhen (Second Affiliated Hospital of Southern University of Science and Technology), Shenzhen, Guangdong, China; 2 Environment and Health Department, Shenzhen Center for Disease Control and Prevention, Shenzhen, Guangdong, China; 3 Department of Healthcare-Associated Infection Management, Shenzhen Maternity and Child Healthcare Hospital, Southern Medical University, Shenzhen, Guangdong, China

*To the Editor—*The novel coronavirus SARS-CoV-2 causes a severe acute respiratory disease named coronavirus disease 2019 (COVID-19) by the World Health Organization (WHO). It was first identified by Chinese scientists in December 2019.^1,2^ Infection with this virus occurs through human-to-human transmission, like SARS and MERS. It has been reported that COVID-19 can spread through droplets, aerosols, skin-to-skin contact or digestive tract.^3,4^ By March 1, 2020, >80,000 cases had been confirmed in China; meanwhile, >7,200 cases had been diagnosed in the other 61 countries, including Korea, Iran, the United States, and elsewhere.^5^

As a megalopolis with a large floating population, the epidemic situation in Shenzhen developed rapidly.^6^ On January 11, the first case was confirmed in Shenzhen; it was also the first case in Guangdong Province.^7^ By March 1, a total of 418 cases had been confirmed in just 50 days. In this outbreak, the Third People’s Hospital of Shenzhen has been the only designated hospital for COVID-19 patients in Shenzhen. From January 11 through March 1, the average number of confirmed patients per day was 18.44 ± 16.18 (Fig. 1). The maximum number of admitted patients was 56 for 1 day. Among them, the average number of severely ill patients was 4.04 ± 5.10 and the average number of critically ill patients per day was 0.84 ± 2.23. The peak 1-day maximum number of severely ill patients was 17 and the peak 1-day maximum number of critically ill patients was 12. In total, 31 infectious diseases areas, 508 wards, and 1,374 hospital beds were applied for COVID-19 patients.

**Fig. 1.**
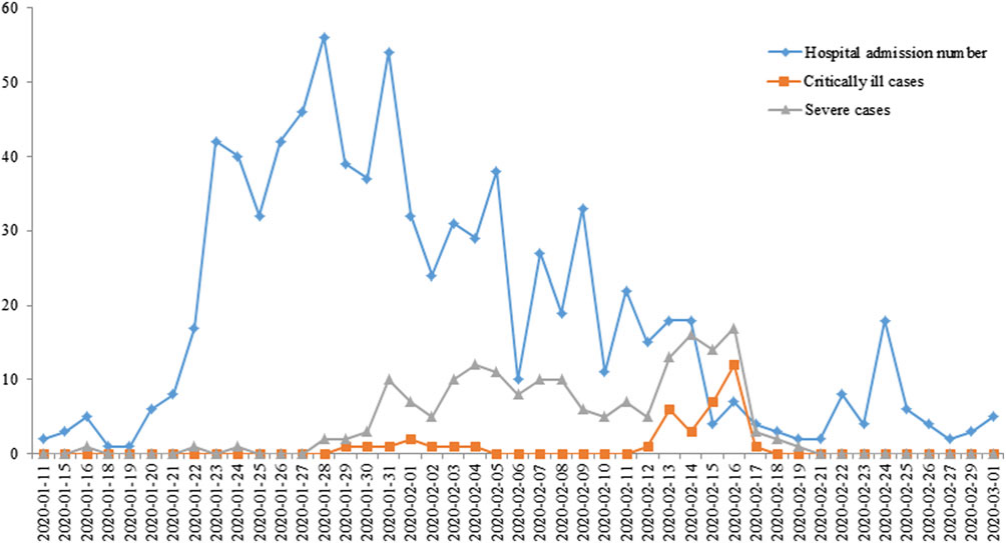
The number of patient admission of the Third People’s Hospital of Shenzhen from January 11 through March 1, 2020.

Facing this sudden epidemic, the infection prevention and control department of the Third People’s Hospital of Shenzhen did a great deal of work in the prevention of virus transmission, cross infection, and medical staff infection.^8^ They initiated the first fever screening system, which has played an important role in the prevention and control of hospital infection.

## Three levels of triage for fever patients

To avoid the cross infection of patients in outpatient treatment, the hospital strictly controls entry to and exit from the outpatient area. The outpatient hall and emergency hall each have only 1 entrance and 1 exit. Upon arrival, all patients need to go through “3 passes.” The first pass is the pre-examination and triage stage where doctors and nurses take temperature measurements and do a triage evaluation. The epidemiological history and clinical symptoms of the patients are carefully collected by the triage personnel. Each fever patient is issued a surgical mask and provides registration details traceability. According to the specific content of the questionnaire, an Healthy-QR color code is set as red, yellow, or green. Patients assigned a red code are escorted to the fever clinic by special staff on a designated route. The yellow code is used to alert the follow-up clinic after the discharge of a COVID-19 patient. A green code provides the patient entry to the outpatient hall. The second pass is a specialized triage pass. The patient first presents the Healthy-QR color code assigned in the first pass. The triage nurse takes the patient’s temperature again and acquires an oral epidemiological history. In the third triage pass, the patient enters a consulting room, where a doctor signs off on the patient’s written epidemiological history and the patient’s medical history. These two signed notices are then filed. In the second and third triage passes, the patients identified with fever at the pre-examination are delivered to the fever clinic along a designated route by assigned staff from the pre-examination triage. The areas that the patients pass through are disinfected immediately.

**Fig. 2.**
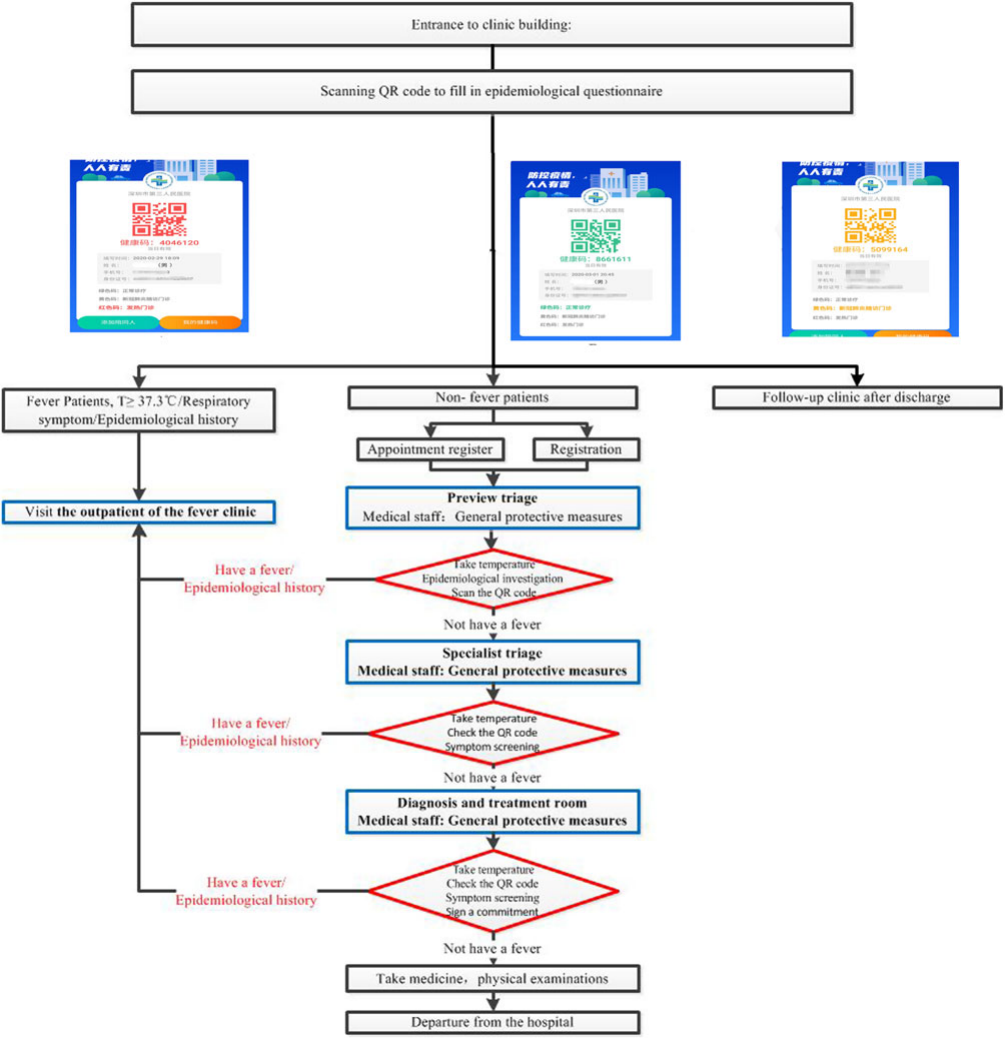
The flowchart for the 3-stage triages system and Healthy-QR codes. The blue boxes represent the triage table or consulting room, and the red boxes represent the inspection method.

The flowchart for pre-examination and the 3-stage triage system are shown in Fig 2. From January 11 to March 1, a total of 421 people went to the fever clinic under the oversight of the outpatient pre-examination triage office. Among them, 12 patients were confirmed to have COVID-19. The strict 3-level triage prevents COVID-19 patients from infecting other patients or medical staff in the public area of the clinic.

## District management of fever clinics

**Fig. 3.**
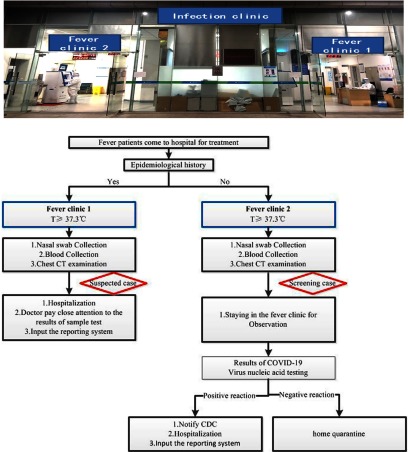
The flowcharts for fever clinics. The blue boxes represent the consulting room and the red boxes represent the patient classification.

The fever clinic is located in an independent area far from the clinic hall. The patient hallway and the medical staff hallway are independent and do not intersect. The fever clinic comprises consulting rooms, a waiting area, a charge office, a pharmacy, a specimen collection office, a x-ray examination area, and resuscitation rooms. To our knowledge, this is the first time a fever clinic has been divided into different areas. The 2 fever clinics are relatively independent and do not overlap. Patients are screened and assigned a Healthy-QR color code (Fig. 3). The red Healthy-QR code indicates that the patient has an epidemiological history and that the patient should go to fever clinic 1 for treatment. The green Healthy-QR code indicates that the patient has no epidemiological history, and the patient should go to fever clinic 2 for treatment.

As shown in Table 1, the fever clinics received 2,140 visits from January 24 to March 1. Among them, 1,408 patients were admitted to fever clinic 1, and all patients were given a nucleic acid test (NAT) for SARS-Cov-2. In addition, 56 patients were NAT positive, and the NAT-positive rate for SARS-CoV-2 was 3.98%.

**Table 1.** Comparison of Nucleic Acid Test (NAT) Results Between 2 Fever Clinics

Location	Total Visits	No. of Patients With NAT	No. of Confirmed Patients	Positive Rate, %	*P* Value
Fever clinic 1 (with epidemiological history)	1,408	1,408	56	3.98	.001
Fever clinic 2 (without epidemiological history)	732	732	2	0.27	

Meanwhile, 732 patients were admitted to fever clinic 2, and all patients were tested for the NAT. Among them, 2 cases were NAT positive, and the NAT- positive rate was 0.27%. The difference in NAT-positive rates between the 2 fever clinics was statistically significant (χ^2^ = 25.059; *P* < .001). Thus, we conclude that this method effectively prevents cross infection of patients in the fever clinic.

Our hospital has made great contributions to both the treatment of patients and the prevention of the spread of this epidemic. By March 1, a total of 418 cases of COVID-19 had been admitted to this hospital, and 163 cases had been discharged. Meanwhile, none of the 1,264 medical staff had been infected. Moreover, no cross infection had occurred among the 1, 870 other patients hospitalized during the same period.

Reviewing the cross infections and medical staff infections in other hospitals, we offer our hypotheses for several main reasons for cross infection in hospitals. First, the patients did not undergo strictly screening and triage before treatment. Second, the division of treatment zones between suspected patients and ordinary patients were not clear. Third, suspected patients were neglected and personal protective equipment of medical staff was inadequate in the early stage of the epidemic.

We offer our experience regarding the fever screening system at our institution for the benefit of other hospitals involved in the treatment of patients infected with COVID-19. We believe that comprehensive victory over the new coronavirus pneumonia epidemic is not far away.
